# Vasculitis mimicking immunoglobulin-G4-related disease with involvement of the submandibular and lacrimal glands and periaortitis: A case report

**DOI:** 10.1097/MD.0000000000034492

**Published:** 2023-07-28

**Authors:** Mamika Kuribayashi, Hidesato Odaka, Susumu Takahashi, Takuo Tokairin, Hirokazu Kurokawa

**Affiliations:** a Post Graduate Clinical Education Center, Japanese Red Cross Akita Hospital, Akita, Japan; b Department of Respiratory Medicine, Japanese Red Cross Akita Hospital, Akita, Japan; c Department of Pathology, Japanese Red Cross Akita Hospital, Akita, Japan; d Faculty of Nursing, Japanese Red Cross Akita College of Nursing, Akita, Japan.

**Keywords:** aorta, immunogloburin G4-related disease, lacrimal gland, submandibular gland, vasculitis

## Abstract

Vasculitis does not usually induce glandular tissue lesions. Herein, we present a case of vasculitis with a distribution similar to that of immunoglobulin-G4-related disease (IgG4-RD), including that of the submandibular and lacrimal glands, and periaortitis.

**Case report:**

A 70-year-old man presented with shortness of breath. Laboratory findings revealed an IgG4 level of 191 mg/dL, negative antineutrophil cytoplasmic antibody test, and C-reactive protein level of 8.33 mg/dL. Magnetic resonance imaging of the head and computed tomography of the neck revealed bilaterally enlarged submandibular and lacrimal glands. Neck-to-pelvis computed tomography revealed bilateral infiltrative shadows in the lower lobes of both lungs, mass shadows in both lungs, and periaortitis of the abdominal aorta extending to the common iliac artery. Thus, the patient was diagnosed with IgG4-related respiratory disease and periaortitis/periarteritis. Prednisolone was administered at a dose of 35 mg (0.6 mg/kg daily). The dose was gradually tapered while observing the effects of the treatment. Imaging findings indicated an improvement and the C-reactive protein and IgG4 levels decreased, indicating a successful treatment course. However, after reexamination of the pathological findings, the diagnosis changed from IgG4-RD to vasculitis. One year after treatment initiation, the patient symptoms have stabilized.

**Conclusion:**

Vasculitis can present with lesions and pathological findings similar to those of IgG4-RD.

## 1. Introduction

The diagnosis of immunoglobulin-G4-related disease (IgG4-RD) and vasculitis has been increasing since the novel concept of IgG4-RD was established in Japan in 2011.^[1]^

IgG4-RD frequently affects the pancreas and lacrimal and salivary glands. Vasculitis commonly affects the upper respiratory tract, lungs, kidneys, eyes, and peripheral nerves^[2]^; however, glandular tissue lesions are not considered a typical presentation of vasculitis. Herein, we report a case of vasculitis in the submandibular and lacrimal glands with periaortitis, which presented with a distribution similar to that of IgG4-RD.

## 2. Case presentation

Ethics approval and consent for this case report were waived. We have obtained written consent from the patient to present the case.

A 70-year-old man developed a sore throat and cough while working. He also experienced shortness of breath. He visited a physician 14 days later. Pneumonia was suspected based on his chest radiograph findings; therefore, the patient was referred to another hospital for treatment. Upon admission, a chest computed tomography (CT) was performed which revealed masses in the lower lobes of both lungs. However, no improvement was observed after 1-week of treatment with tazobactam/piperacillin (4.5 g × thrice/day). The patient was referred to our department for further management. He had a history of smoking 20 cigarettes per day for 47 years (20–67 years of age).

On admission, his vital signs were as follows: blood pressure, 117/67 mm Hg; pulse rate, 80 bpm; respiratory rate, 18/min; O_2_ saturation, 96% on room air; and temperature, 36.7°C. The submandibular glands were palpable and approximately 2 cm in size. We did not identify any pulmonary vessel murmurs, abdominal vascular murmurs, or any other remarkable physical findings.

The abnormal laboratory findings upon admission were as follows: The IgG4, IgE, rheumatoid factor, antinuclear antibody, and C-reactive protein (CRP) levels and erythrocyte sedimentation rate were elevated. All other blood investigation results, including the myeloperoxidase-antineutrophil cytoplasmic antibody (ANCA) and proteinase-3-ANCA levels, were within the normal reference range (Table 1).

**Table 1:** Patient laboratory test results.

Laboratory investigation	Results	Reference range
WBC count	5900/μL	3300–8600/μL
Neutrophils	76.6%	41.2–69.7%
Lymphocytes	12.6%	22.1–46.9%
Monocytes	7.7%	4.1–9.6%
Eosinophils	2.2%	0.0–3.5%
Basophils	0.9%	0.0–1.1%
RBC count	482 × 10^4^/μL	386–492 × 10^4^/μL
HGB	13.9 g/dL	11.6–14.8 g/dL
HCT	42.3%	35.1–44.4%
PLT	42.8 × 10^4^/μL	15.8–34.8 × 10^4^/μL
ESR	89 mm/h	≤10 mm/hr
TP	7.4 g/dL	6.6–8.1 g/dL
Alb	3.3 g/dL	4.1–5.1 g/dL
T-Bil	0.3 mg/dL	0.4–1.5 mg/dL
G-GT	101 IU/L	13–64 U/L
AST	17 IU/L	13–30 U/L
ALT	28 IU/L	7–23 U/L
CK	29 IU/L	59–248 U/L
LDH	133 IU/L	124–222 U/L
BUN	16.8 mg/dL	8.0–20.0 mg/dL
Cr	0.71 mg/dL	0.46–0.79 mg/dL
Na^2+^	139 mmol/L	138–145 mmol/L
K^+^	3.9 mmol/L	3.6–4.8 mmol/L
Cl^−^	103 mmol/L	101–108 mmol/L
CRP	8.33 mg/dL	0.00–0.14 mg/dL
IgG	1408 mg/dL	861–1747 mg/dL
IgG4	191 mg/dL	11.0–121.0 mg/dL
IgA	201 mg/dL	93–393 mg/dL
IgM	87 mg/dL	33–183 mg/dL
IgE	1200 IU/mL	0–170 IU/mL
C4	25 mg/dL	11–31 mg/dL
C3	138 mg/dL	73–138 mg/dL
ACE	10.8 IU/mL	7.7–29.4 IU/mL
RF	83 IU/mL	0–15 IU/mL
Anti-nuclear antibody	40 antibody titer	<40 antibody titer
Homogenous pattern	(+)	(−)
Anti-ds-DNA antibody	(−)	(−)
Anti-SS-A antibody	(−)	(−)
Anti-SS-B antibody	(−)	(−)
Anti-CCP antibody	0.8 IU/mL	≤4.5 U/mL
PR3-ANCA	<0.5 IU/mL	≤2.0 IU/mL
MPO-ANCA	<0.5 IU/mL	<0.5 IU/mL
HCV antibody	(−)	(−)
HBs antigen	(−)	(−)
Urinary protein	(−)	(−)
Urinary occult blood	(−)	(−)

ACE = angiotensin-converting enzyme, Alb = albumin, ALT = alanine transaminase, ANCA = antineutrophil cytoplasmic antibody, AST = aspartate aminotransferase, BUN = blood urea nitrogen, CCP = cyclic citrullinated peptide, Cl = chloride, Cr = creatinine, CRP = C-reactive protein, ds-DNA = double stranded deoxyribonucleic acid, ESR = erythrocyte sedimentation rate, HBs = hepatitis B surface, HCT = hematocrit, HCV = hepatitis C virus, HGB = hemoglobin, Ig = immunoglobulin, K = potassium, LDH = lactate dehydrogenase, MPO = myeloperoxidase, Na = sodium, PLT = platelet, PR3 = proteinase-3, RBC = red blood cell, RF = rheumatoid factor, T-Bil = total bilirubin, TP = total protein, WBC = white blood cell.

**Figure 1.**
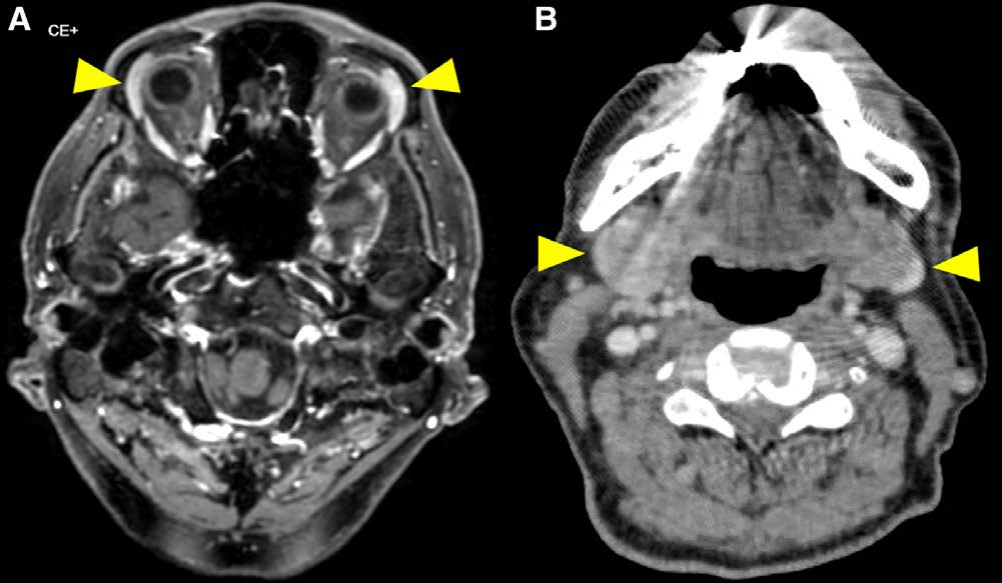
(A) Head MRI revealing swollen lacrimal glands (arrowhead). (B) Neck CT revealing bilaterally enlarged submandibular glands (arrowhead). CT = computed tomography, MRI = magnetic resonance imaging.

**Figure 2.**
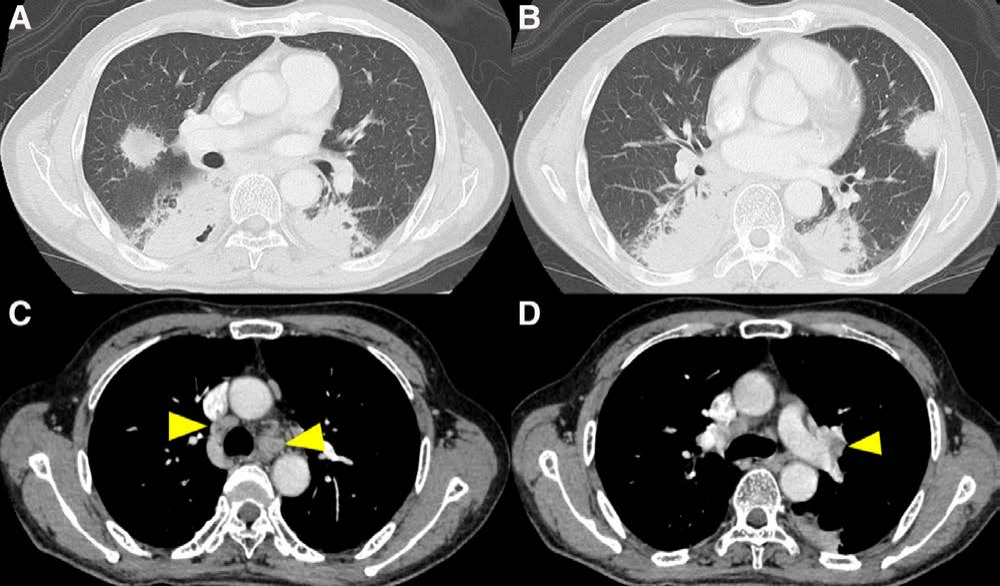
(A and B) Neck-to-pelvis CT revealing bilateral infiltrative shadows in the lower lobes of both lungs, as well as mass shadows in the both lungs. (C and D) Swelling of the bilateral hilar (arrowhead) and mediastinal (arrowhead) lymph nodes. CT = computed tomography.

**Figure 3.**
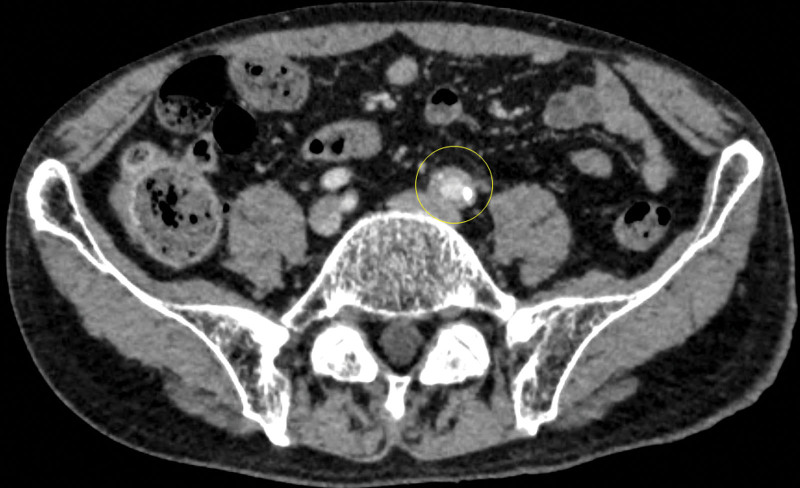
CT findings were suggestive of periaortitis of the abdominal aorta up to the common iliac artery (circle). CT = computed tomography.

Head magnetic resonance imaging and neck CT revealed bilaterally enlarged submandibular and lacrimal glands (Fig. 1A and B). Neck-to-pelvis CT revealed bilateral infiltrative shadows in the lower lobes of both lungs, mass shadows in the middle lobe of right lung and lingula of left lung, swelling of bilateral hilar and mediastinal lymph nodes, and multifocal ground-glass opacities (Fig. 2A–D). Periaortitis of the abdominal aorta extending up to the common iliac artery was also detected on CT (Fig. 3).

**Figure 4.**
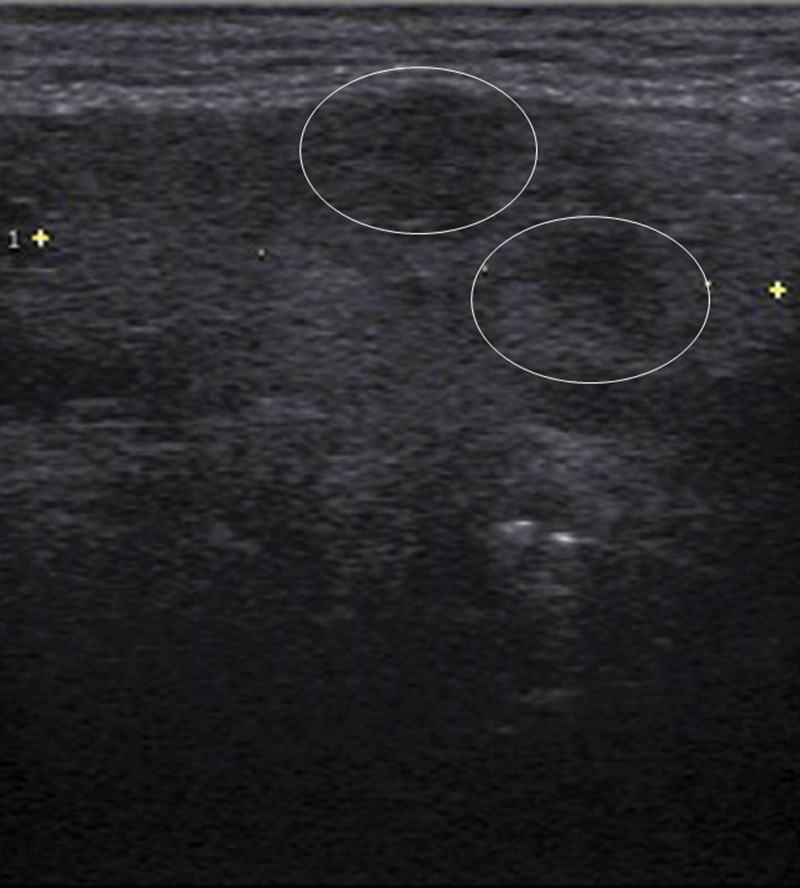
Submandibular gland ultrasonography before treatment revealing coarse hypoechoic areas (circle).

Ultrasonography of the submandibular gland revealed coarse hypoechoic areas (Fig. 4).

Nasopharyngolaryngoscopy revealed no abnormal findings in the paranasal cavity; thus, no biopsy was performed.

**Figure 5.**
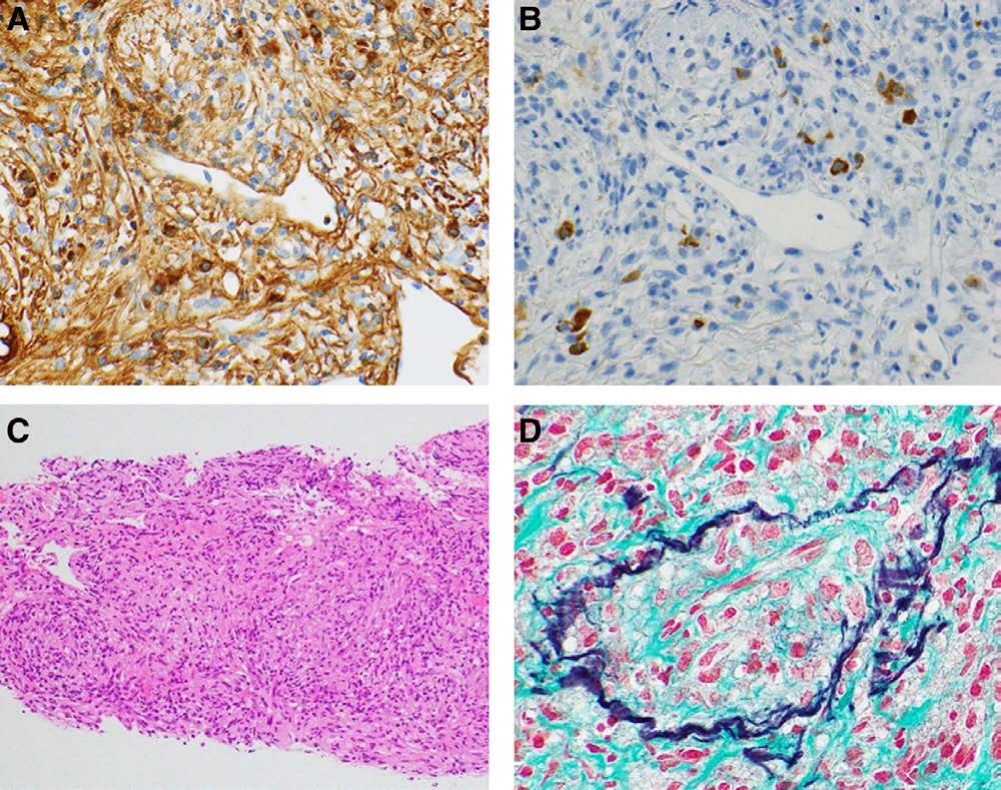
(A) IgG immunohistochemical staining revealing numerous IgG + plasma cells in the left lung (×400). (B) IgG4 immunohistochemical staining revealing >10 IgG4 + plasma cells in one high power field of view in the left lung (×400). The IgG4+/IgG + cell ratio exceeded 40 in some areas. (C) Hematoxylin and eosin staining suggestive of storiform fibrosis in the left lung (×100). (D) Elastica–Masson staining indicative of obliterative phlebitis in the left lung (×400). IgG = immunoglobulin-G.

**Figure 6.**
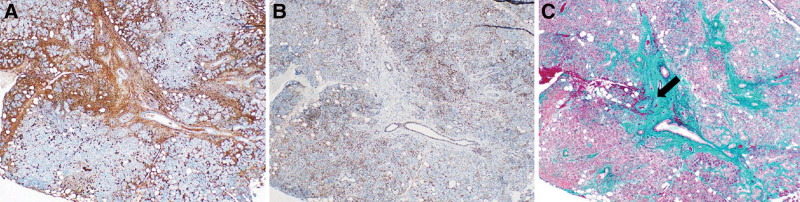
(A) IgG immunohistochemical staining indicating numerous IgG + plasma cells in the submandibular gland (×100). (B) IgG4 immunohistochemical staining revealing > 10 IgG4 + plasma cells in 1 field of view in the submandibular gland (×100). The IgG4+/IgG + cell ratio is >40. (C) Elastica–Masson staining revealing less obliterative phlebitis (arrow) in the submandibular gland (×100). IgG = immunoglobulin-G.

We performed a CT-guided biopsy of the mass in the left lung; histological analysis revealed dense lymphocytic and plasmacytic infiltration with fibrosis. Immunohistochemical analysis revealed numerous IgG4 + cells in the mass (≥50 cells/high-power field); in some areas, the IgG4+/IgG + cell ratio exceeded 40. Storiform fibrosis and obliterative phlebitis were also observed (Fig. 5A–D). We biopsied the submandibular gland; histological analysis revealed >10 IgG4 + plasma cells in 1 field of view, and the IgG4+/IgG + cell ratio was <40. Minimal obliterative phlebitis was observed (Fig. 6A–C). Based on the diagnostic criteria for IgG4-RD,^[3]^ the patient was diagnosed with IgG4-related respiratory disease and periaortitis/periarteritis.

Prednisolone was administered at a dose of 35 mg (0.6 mg/kg daily). The dose was gradually tapered while observing the effects of the treatment. The subsequent improvement in imaging findings and decrease in CRP and IgG4 levels, indicated a successful treatment course. There was no specialist in IgG4-RD in our hospital. Thus, we reviewed the samples collected before diagnosis; we did not repeat the biopsy. The diagnosis was changed from IgG4-RD to vasculitis.

**Figure 7.**
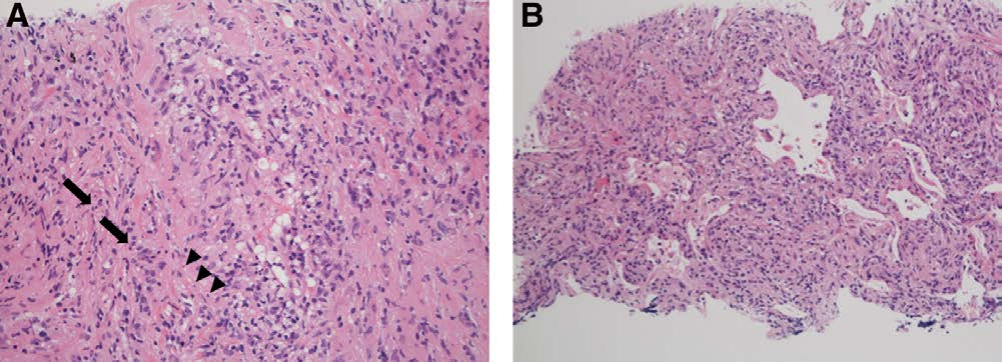
(A) The vascular endothelium is damaged, and cell debris (arrowhead) from nuclei is observed, suggesting vasculitis in the lung tissue. Neutrophils (arrow) are present in the lung (×400). (B) Histological analysis of the lung tissue (×100). This area should show normal alveolar structure; however, the main interstitium is inflamed, the alveolar epithelium is damaged, and exudate is present in the lumen. These findings are usually not observed in patients with IgG4-RD. IgG4-RD = immunoglobulin-G4-related disease.

**Figure 8.**
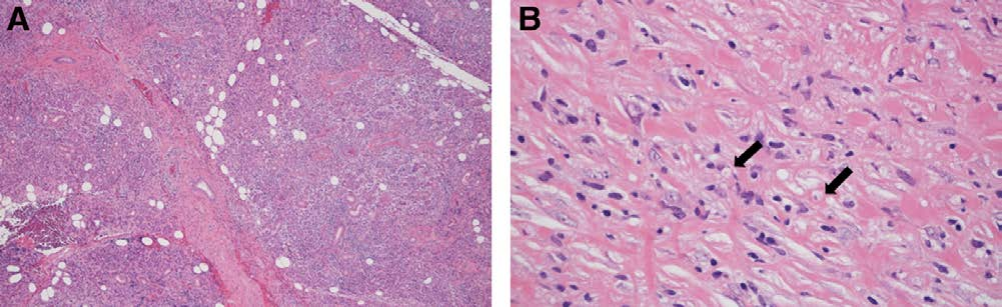
(A) Lymphocytes and plasma cells diffusely infiltrate into the lobular and submandibular gland tissue (×100). (B) Neutrophils and cell debris (arrow) are observed in the interlobular septa (×400).

The lung tissue revealed damaged vascular endothelium, some nuclei debris, and neutrophilic infiltration, suggesting vasculitis (Fig. 7A). The alveolar epithelium was damaged, and an exudate was present in the lumen (Fig. 7B). In the submandibular gland tissue, lymphocytes and plasma cells had diffusely infiltrated the lobules (Fig. 8A), resembling IgG4-RD. Neutrophils and cell debris were also observed in the interlobular septa (Fig. 8B). These findings are not observed in patients with IgG-RD. The IgG4+/IgG + cell ratio was also low.

One and a half years after treatment initiation, the symptoms have stabilized; therefore, the remission is being maintained with a small dose of prednisolone. If symptoms worsen in the future, we will consider the use of immunosuppressive drugs in combination with prednisolone.

## 3. Discussion

We identified 2 important clinical issues in this case. First, vasculitis can present with a distribution similar to that of IgG4-RD. Second, submandibular gland ultrasonography may be useful for distinguishing between IgG4-RD and vasculitis.

The vasculitis in this case may have been ANCA-negative granulomatosis with polyangiitis (GPA). The concept of ANCA-negative ANCA-associated vasculitis (AAV) was advocated in the 2012 Chapel Hill Consensus Conference (CHCC)^[4]^; it discussed that ANCA-negative AAV is analogous to seronegative lupus or rheumatoid arthritis. It is diagnosed if the patient meets the criteria for AAV, but the serological tests for ANCA are negative. In Japan, GPA is diagnosed based on the diagnostic criteria proposed by the Japanese Ministry of Health, Labor and Welfare.^[5]^ Although this case was not definitively GPA, it could probably have been GPA based on these criteria. Additionally, it could not be classified as GPA according to the American College of Rheumatology classification criteria of 1990.^[6]^ However, owing to the histological compatibility with the CHCC criteria of microscopic polyangiitis and radiographic evidence of fixed pulmonary infiltrates present for >1 month, this case was classified as GPA based on the European Medicines Evaluation Agency vasculitis classification algorithm.^[7]^ The 2012 CHCC also states that when patients with GPA exhibit clinical and pathologic changes identical to those observed in respiratory diseases they should remain in the GPA category.

The patient had a lesion in the submandibular gland. Involvement of the submandibular gland in vasculitis is rare.^[8–11]^ The submandibular gland lesion in this case was pathologically similar to that of IgG4-RD; however, cell debris is not typically observed in IgG4-RD, and the IgG4/IgG cell ratio was low. Moreover, parotid involvement in vasculitis is rare.^[12–21]^ Thus, the relationship between salivary gland involvement and vasculitis remains unclear. Additionally, the patient had a lacrimal gland lesion, which has been reported in patients with vasculitis.^[22–24]^

A periaortic lesion was observed in this patient. The CHCC 2012 criteria classifies GPA as a small-vessel vasculitis. Aortic involvement in GPA is rare, and only a few cases have been reported.^[25–35]^ The mechanism of large-vessel involvement in GPA is yet to be elucidated. Histological and clinical observations in previous studies have suggested that large-vessel involvement belongs to the AAV spectrum rather than overlapping with other large-vessel vasculitis.^[35,36]^

The CRP levels in patients with IgG4-RD do not increase significantly^[37]^; however, high CRP levels can be a hallmark of IgG4-related periaortitis/periarteritis.^[38]^ The median CRP level of IgG4-related periaortitis/periarteritis is 6.72 (2.14–24.65) mg/dL.^[39]^ In this case, the CRP levels was 8.33 mg/dL; therefore, we misattributed this elevation to IgG4-related periaortitis. This is a diagnostic limitation of the present study.

Table 2 presents the previously reported cases of GPA with salivary gland involvement.^[8–21]^ GPA with periaortitis was not reported in any of these case reports. Table 3 lists the previously reported cases of GPA with periaortitis.^[25–35]^ Among these, only 1 case of GPA reported a glandular tissue lesion; proteinase-3-ANCA was positive in this case.

**Table 2:** Cases of granulomatosis with polyangiitis with salivary gland involvement.

Yr	Age (yr)	Salivary gland affected	Other organs affected	ANCA	Tissue used for confirmation of diagnosis
author	sex				
1993 Vanhauwaert BG^[8]^	60 W	Submandibular	Nose Kidney	PR3-ANCA	Submandibular gland
2004 Chegar BE^[12]^	54 M	Parotid	Nose Lung Kidney Nerve	PR3-ANCA	Parotid gland
2005 Jones GL^[13]^	59 M	Parotid	Eye	Negative-ANCA	Parotid gland
2008 Frantz MC^[14]^	71 W	Parotid	Lung	PR3-ANCA	Lung
2008 Yamamoto M^[15]^	62 M	Parotid	Skin Nose	MPO-ANCA	Nose
2009 Geyer M^[16]^	69 W	Parotid	Nose Lung	PR3-ANCA	Sinus
2010 Holl-Ulrich K^[17]^	72 W	Parotid	Liver Lung Skin	Negative-ANCA	Parotid gland
2010 Gassling V^[9]^	68 M	Submandibular Parotid	Kidney	PR3-ANCA	Clinical
2011 Almuhaideb A^[18]^	26 M	Parotid	Nose Lung Prostate	PR3-ANCA	Parotid gland
2013 Kenis I^[19]^	60 M	Parotid	Kidney	PR3-ANCA	Parotid gland
2013 Ceylan A^[20]^	37 W	Parotid	Nose Lung	PR3-ANCA	Parotid gland
2014 Schaller T^[10]^	43 M	Submandibular	-	PR3-ANCA	Submandibular gland
2016 Kikuchi R^[11]^	65 M	Submandibular	Nose Lung Skin	PR3-ANCA	Skin
2019 Chouhan A^[21]^	51 M	Parotid	-	PR3-ANCA	Clinical
2023 Kuribayashi M	70 M	Submandibular	Lacrimal gland Lung Aorta	Negative-ANCA	Lung Submandibular gland

ANCA = antineutrophil cytoplasmic antibody, M = man, MPO = myeloperoxidase, PR3 = proteinase-3, W = woman.

**Table 3:** Cases of granulomatosis with polyangiitis with periaortitis involvement.

Yr	Age (yr)	Glandular tissue affected	Other organs affected	ANCA	Tissue used for confirmation of diagnosis
author	sex				
2000 Blockmans D^[25]^	Unknown	-	Nose Lung Nerve Kidney	PR3-ANCA	Aortic
2002 de RS^[26]^	47 M	-	Lung	PR3-ANCA	Lung
2005 Carels T^[27]^	63 M	-	-	MPO-ANCA	Aortic
2006 Levin A^[28]^	61 M	-	Ear Lung	PR3-ANCA	Lung
2009 Minnee RC^[29]^	51 M	-	Testis Nerve Skin	PR3-ANCA	Testis
2011 Kasagi S^[30]^	61 W	-	Ear Lung Spinal	MPO-ANCA	Clinical
2016 González Revilla EM^[31]^	74 M	-	Lung	PR3-ANCA	Lung Aortic
2017 Miyawaki M^[32]^	60 M	-	Lung Heart	PR3-ANCA	Clinical
2018 Kaga H^[33]^	69 M	-	Eye Nose Lung Kidney	MPO-ANCA	Eye Kidney
2018 Kaushik P^[34]^	77 M	Submandibular	Lung	PR3-ANCA	Aortic Lung Submandibular gland
2021 Cho U^[35]^	27 M	-	Lung	Positive-ANCA	Lung
2023 Kuribayashi M	70 M	Submandibular Lacrimal	Lung	Negative-ANCA	Lung Submandibular gland

ANCA = antineutrophil cytoplasmic antibody, M = man, MPO = myeloperoxidase, PR3 = proteinase-3, W = woman.

To the best of our knowledge, this is the first report of vasculitis with a distribution of lesions similar to that of IgG4-RD, including the submandibular and lacrimal glands, and association with periaortitis.

Submandibular gland ultrasonography may be useful in distinguishing IgG4-RD from vasculitis. Characteristic ultrasonographic changes in the submandibular gland of patients with IgG4-RD are as follows: hypoechoic areas in a nodal pattern with high vascularity and/or superficial hypoechoic areas in a reticular pattern. The sensitivity, specificity, and accuracy of ultrasonographic diagnosis of IgG4-RD are 100%, 83.8%, and 91.2%, respectively.^[40]^ In this case, coarse hypoechoic findings were observed, which differ from the ultrasonographic findings in IgG4-RD; this was useful for the diagnosis. Currently, IgG4-RD cannot be completely ruled out based on ultrasonographic findings alone; however, diagnosis can be made in combination with serological findings. Our study findings need to be externally validated at multiple facilities.

## 4. Conclusion

Vasculitis can present with a distribution similar to that of IgG4-RD, and ultrasonography of the submandibular gland may be useful in distinguishing between IgG4-RD and vasculitis. Unawareness of this, could lead to misdiagnoses. Therefore, ultrasonography of the submandibular gland should be performed frequently. Further studies should be conducted to determine the occurrence of conditions mimicking IgG4-RD and whether routine submandibular gland ultrasonography may contribute to the diagnosis.

## Acknowledgments

We would like to thank the Japanese Association for IgG4-related Diseases for reexamining the pathological findings.

## Author contributions

**Writing – original draft:** Mamika Kuribayashi, Hidesato Odaka.

**Writing – review & editing:** Susumu Takahashi, Takuo Tokairin, Hirokazu Kurokawa.
